# An Evolution of the “Golden Age” of Dermatologic Surgery (1958-2008)

**DOI:** 10.4103/0974-2077.41147

**Published:** 2008-01

**Authors:** Lawrence M. Field

**Affiliations:** *MD, FIACS, International Travelling Chair of Dermatologic Surgery (International Society of Dermatologic Surgery) and Professor Emeritus, University of California, San Francisco (Dermatologic Surgery), 2006 and Stanford University, Emeritus Fellow and Examiner, American Board of Cosmetic Surgery, Senior Consulting and Contributing Editor, Dermatologic Surgery*

To the Readership *Journal of Cutaneous and Aesthetic Surgery*

I am privileged to render my compliments on the success in launching your new journal in India. I wish you all great success and a very significant impact on the subspeciality in your country.

The undersigned has lived through what will become “The Golden Age of Dermatologic Surgery”; a half century spanning the period from 1958 to present. I am presenting here what I see as the major events and trends for Dermatologic Surgery. I present them for your perusal and then your urgent and deep thoughts:

- Continued “Femalization” - 62% of all dermatology residents in USA are women. Thirtyeight percentage of all American Academy of Dermatology (AAD) members are women. Fifty years ago, it was 2-3%!
- Dermatology is a preferred speciality for young doctors. “Top medical students pick dermatology”(President-Elect's Plenary Session at AAD, February 3, 2008).
- Marginalization of speciality as related to other specialities.
- Evolution of “Cosmoceuticals” (there is a lot of hype, with little or no standards).
- Less invasive surgery is becoming increasingly popular. This leads to trivialization of procedures. Practitioners of other specialities, general practitioners (GPs) and even cosmeticians are now attempting some of these procedures.
- More cosmetic surgeries are being performed and there is a fear that these procedures are receiving undue and unwarranted attention of young doctors.
- Fillers are being talked about, heard and used more and more; Fillers, fillers, fillers, fillers (and wrinkles).
- This is the age of combination therapies; for synergism, rejuvenation, facial shaping etc.
- Expanding technologic incorporations everywhere. This has lead to increasing commercialization.
- All these mean that dermatology in general and aesthetic dermatology and surgery in particular have become more and more lucrative. Will all this come at the cost of traditional dermatology?
- More teaching work is being done at workshops: workshops, workshops everywhere. Most workshops are expensive.
- Mentorships are increasingly popular; skills are being learnt from practicing dermatologists and not just teaching institutions.
- Trivialization of procedures is leading to incorporation/inclusion of home treatments (e.g. hair laser).
- Invasion of offices, hospitals with methoxicillin resistant *Staphylococcus aureus* and drug resistant tuberculosis.
- Public safety campaigns are commonplace (we as a speciality are better and safer than “they” are).
- Continued “turf” invasion by general and plastic surgeons.
- Continued law making and practice limiting adversity from plastic surgeons and anaesthesiologists.
- Contamination by paid investigators, payments to our societies by companies and the “privilege” of presentations at our meetings are of real concern.
- Increasing number of dermatologists now work in private offices, leading to a continuing separation of physicians who affiliate with hospitals, from those in private offices (Practice Trends, *Skin and Allergy News* February 2008)
- Mesotherapy spas (“Lipo dissolve”) are facing closure and bankruptcy.
- Mesotherapy is illegal in some states now and legally challenged in many states in USA. American Academy of Dermatology (AAD) and American Society for Dermatologic Surgery (ASDS) consider these approaches inappropriate and unproven.
- Other important and relevant issues include cost-efficiency, communication costs of cell phone, pager, phone, internet access, coding problems, electronic billing, workshops and online bill payments (time, postage, checks, trips to post office). (Lynott, W, Welcoming 2008, Practice Management, *Dermatology Times*, February 2008).

Well, these are all issues needing thought and consideration. I hope, I have lighted some fire as we bridge from the past through now to the future. Good luck!

